# Function of capric acid in cyclophosphamide-induced intestinal inflammation, oxidative stress, and barrier function in pigs

**DOI:** 10.1038/s41598-017-16561-5

**Published:** 2017-11-28

**Authors:** Sang In Lee, Kyung Soo Kang

**Affiliations:** 1 Department of Animal Resource and Science, Dankook University, Cheonan, Chungnam 330-714 Republic of Korea; 2 Bio Division, Medikinetics, Inc., Hansan-gil, Pyeongtaek-si, Gyeonggi-do, 17792 Republic of Korea

## Abstract

The small intestine is not only critical for nutrient absorption, but also serves as an important immune organ. Medium-chain fatty acids have nutritional and metabolic effects and support the integrity of the intestinal epithelium. However, their roles in intestinal immunity in pigs are not fully understood. We investigated the effects of a medium-chain fatty acid, capric acid, on intestinal oxidative stress, inflammation, and barrier function in porcine epithelial cells and miniature pigs after treatment with the immune suppressant cyclophosphamide. Capric acid alleviated inflammatory cytokine production (TNF-α and IL-6) and related gene expression (*NF-κB*, *TNF-α*, *IFN-γ*), alleviated oxidative stress (GSSG/GSH ratio, H_2_O_2_, and malondialdehyde), and increased oxidative stress-related gene expression (*SOD1* and *GCLC*) in cyclophosphamide-treated IPEC-J2 cells. The permeability of FD-4 and expression of *ZO-1* and *OCLN* in cyclophosphamide-treated IPEC-J2 cells were reduced by capric acid. Dietary capric acid reduced TNF-α, IL-6, and MDA levels and increased SOD, GPx, and the expression of genes related to pro-inflammatory, oxidative stress, and intestinal barrier functions in cyclophosphamide-treated miniature pigs. These results revealed that capric acid has protective effects against cyclophosphamide-induced small intestinal dysfunction in pigs.

## Introduction

The gastrointestinal (GI) tract is not only important for the digestion, absorption, and metabolism of nutrients, but also serves as a critical immune organ, since it has the most immune cells in the body^1^. Pathogenic or nonpathogenic challenges activate the GI immune system, leading the production of a diverse set of specialised cells and signalling molecules, especially pro-inflammatory cytokines, such as tumour necrosis factor (TNF)-α, interleukin (IL)−1β, and IL-6, resulting in intestinal mucosal injury and dysfunction via the over-production of these cytokines^2,3^. In pigs, intestinal infections drastically amplify inflammatory responses, affecting the intestinal morphology, mucosal functions (including nutrient digestion and absorption), and intestinal barrier function, resulting in reduced feed intake, weight gain, and an altered gain/feed ratio^4–7^. In addition to intestinal pathogenic infection, intestinal inflammation can be induced by stress, such as weaning and oxidative stress^3,8^.

Oxidative stress caused by an imbalance between pro-oxidants and anti-oxidants is associated with the excessive production of reactive oxygen species (ROS) and an antioxidant deficiency^9^. In the small intestinal epithelium, oxidative stress is a major cause of barrier malfunction and the pathogenesis of various gastrointestinal diseases, such as enterocolitis, gastrointestinal cancers, celiac disease, and inflammatory bowel disease^10–12^. The small intestinal epithelium serves as an important part of the first line of defence against pathogens and regulates the absorption of solutes and macromolecules, including nutrients. The intestinal barrier is composed of a single layer of columnar epithelial cells sealed by junctional complexes including, tight and adherens junctions, in close proximity to the apical and lateral side of the paracellular space. These structures are affected by oxidative stress, since the pathophysiology of a redox imbalance is characterised by disrupted junctional complexes^13–15^. Disruption of the junctional complexes can facilitate the passage of macromolecules and pathogens through the intestinal epithelium into the body^16–18^. Oxidative stress also affects mitosis, apoptosis, and differentiation in small intestinal epithelial cells from the crypt to the villus.

Fatty acids, used as feed additives, are important components of the cell membrane, cell-signalling molecules, metabolic substrates in many biochemical pathways, and immune modulators^19^. Medium-chain fatty acids (MCFAs), with aliphatic tails of six to twelve carbon atoms, have specific nutritional and metabolic effects, including rapid digestion, passive absorption, and obligatory oxidation^8,20^. MCFAs also support the integrity of the intestine, increasing the length of villi and reducing the crypt depth in the small intestine^21–23^. In addition, studies on the effects of various MCFAs such as capric acid and caprylic acid, on the intestinal epithelium and inflammation have indicated that these MCFAs have roles or effects^24,25^. However, little is known about the impact of capric acid, an MCFA, on the physiological function of the small intestine in pigs. Therefore, it is important to study the effect of individual MCFAs, such as capric acid, on the intestinal epithelium and the integrity of the intestine. In the present study, we investigated the functions of capric acid in small intestinal epithelial cells after cyclophosphamide treatment in pigs. To our knowledge, this is the first study of the effect of capric acid on intestinal oxidative stress, inflammation, and barrier function in porcine epithelial cells and miniature pigs.

## Results

### Viability of intestinal epithelial cells after capric acid and cyclophosphamide treatment

**Figure 1:**
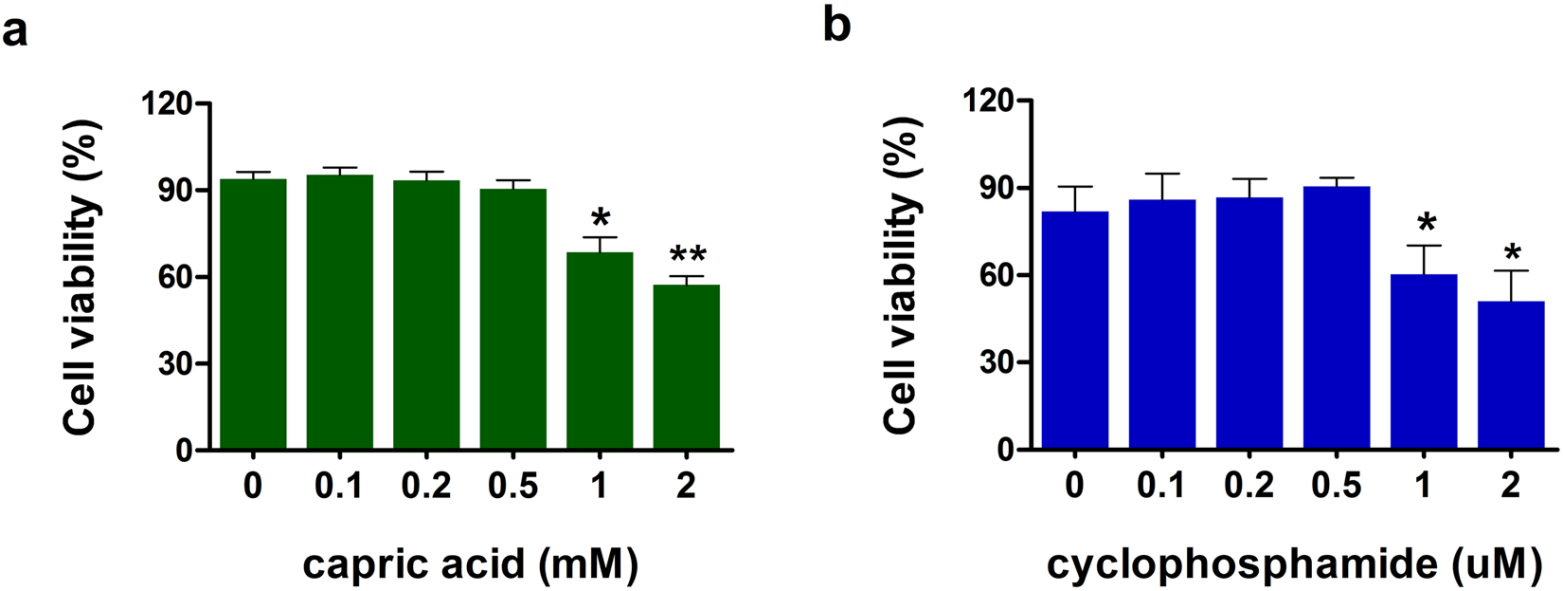
Cytotoxicity of capric acid and cyclophosphamide in IPEC-J2 cells. Cell viability was determined by MTT assays. (**a**) IPEC-J2 cells were incubated with capric acid (0–2 mM) for 24 h. (**b**) IPEC-J2 cells were incubated with cyclophosphamide (0–2 µM) for 1 h. Error bars indicate the standard error of the mean (*n* = 3). Significant differences between control and treatment groups are indicated as **p < 0.01 and *p < 0.05.

To determine the appropriate dose of capric acid and cyclophosphamide for small intestinal epithelial cells, the viability of IPEC-J2 cells was monitored. Exposure to 1 µM CTX (p < 0.05) for 1 h decreased the viability of IPEC-J2 cells (Fig. 1a). Pre-treatment with 1 mM capric acid decreased the viability of IPEC-J2 cells (Fig. 1b). Based on these results, capric acid at 500 µM and cyclophosphamide at 500 µM applied for 1 h were considered safe and were used for subsequent experiments.

### Effect of capric acid on inflammatory markers after cyclophosphamide treatment

**Figure 2:**
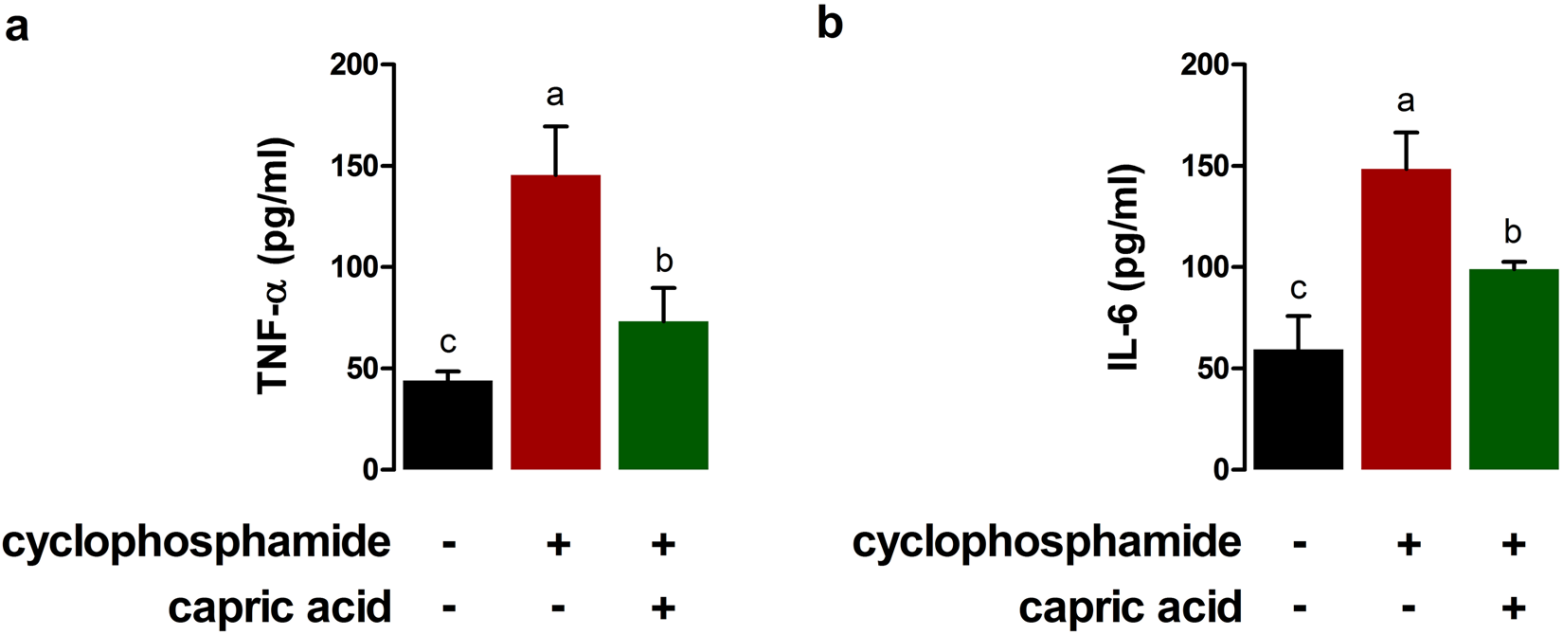
Effects of capric acid treatment on the production of the inflammatory cytokines (**a**) TNF-α and (**b**) IL-6 after cyclophosphamide treatment in IPEC-J2 cells. Concentrations of TNF-α and IL-6 in the cell supernatants were detected by ELISA. Mean cytokine concentrations from several independent experiments are presented (*n* = 3). Error bars indicate the standard error of the mean. A p-value of < 0.05 was considered statistically significant. Lowercase letters (a,b,c) indicate significant differences between treatments based on Duncan multiple range tests.

The effects of capric acid on immune reactions to cyclophosphamide treatment in IPEC-J2 cells were examined. A significantly higher TNF-α concentration was observed in IPEC-J2 cells after treatment with cyclophosphamide than in controls (p < 0.01) (Fig. 2a). Pre-treatment with capric acid significantly reduced the TNF-α concentration after cyclophosphamide of treatment in IPEC-J2 cells (p < 0.05) (Fig. 2a). A significantly higher IL-6 concentration was observed in IPEC-J2 cells after cyclophosphamide treatment than in control cells (p < 0.05) (Fig. 2b). Pre-treatment with capric acid significantly reduced the IL-6 concentration after treatment with cyclophosphamide in IPEC-J2 cells (p < 0.05) (Fig. 2b).

**Figure 3:**
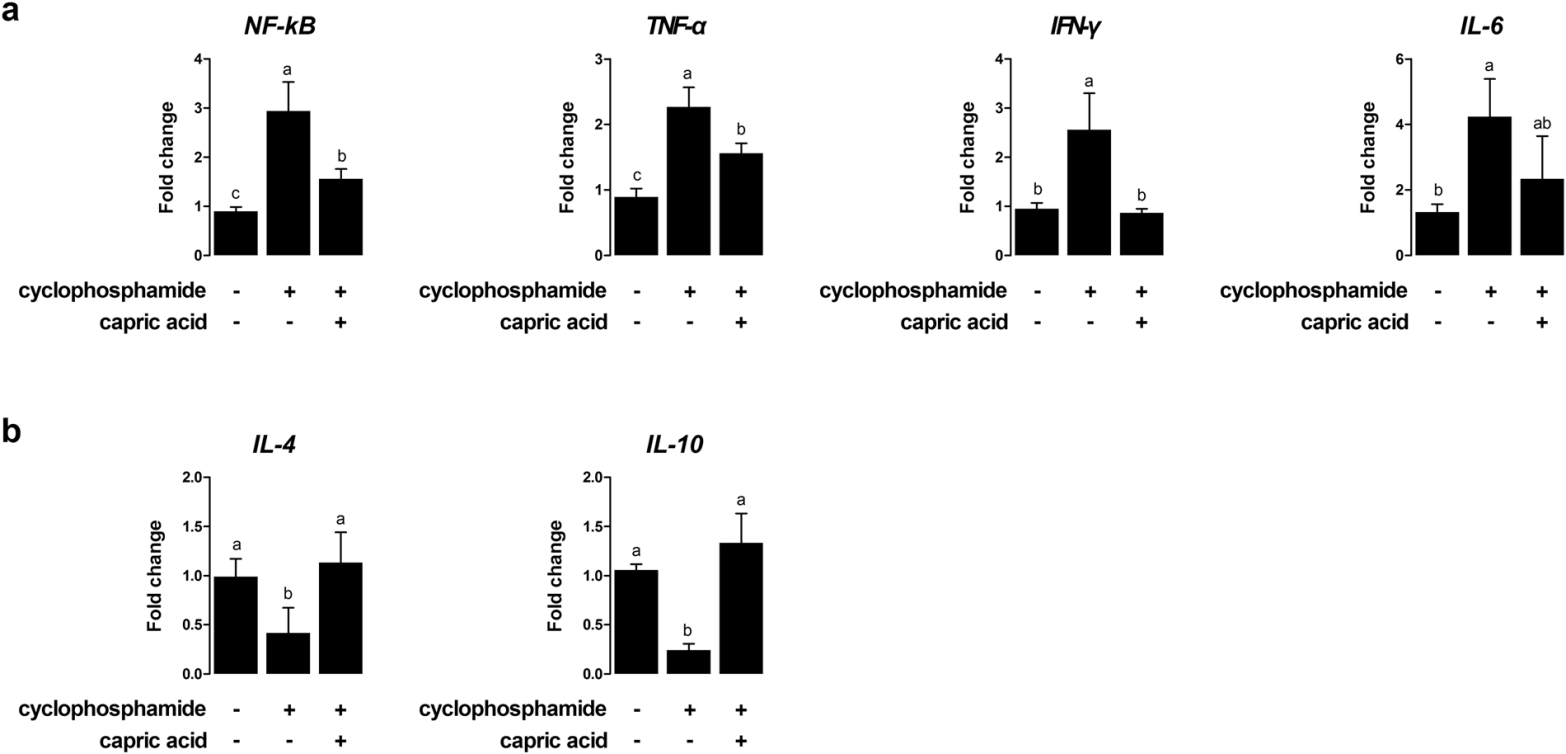
Relative quantitative expression of genes encoding (**a**) pro-inflammatory cytokines (TNF-α, IFN-γ, and IL-6) and (**b**) anti-inflammatory cytokines (IL-4 and IL-10) after capric acid treatment and cyclophosphamide treatment in IPEC-J2 cells. The qRT-PCR data were normalised to the expression of *GAPDH* as an endogenous control gene and calculated using the 2^−ΔΔCt^ method (*n* = 3). Error bars indicate the standard error of the mean. A p-value of <0.05 indicated statistical significance. Lowercase letters (a, b, c) indicate significant differences between treatments based on Duncan multiple range tests.

After cyclophosphamide treatment, the relative expression levels of pro-inflammatory genes, such as *NF-κB* (p < 0.01), *TNF-α* (p < 0.05), *IFN-γ* (p < 0.01), and *IL-6* (p < 0.01) were significantly higher than the expression levels in control cells (Fig. 3a). Pre-treatment with capric acid resulted in reduced *NF-κB* (p < 0.05), *TNF-α* (p < 0.05), and *IFN-γ* (p < 0.05) mRNA levels after treatment with cyclophosphamide in IPEC-J2 cells (Fig. 3a). The levels of *IL-4* (p < 0.05) and *IL-10* (p < 0.001), anti-inflammation genes, were significantly higher in capric acid-treated cells than in control cells after cyclophosphamide treatment (Fig. 3b). Pre-treatment with capric acid increased the expression of *IL-4* (p < 0.05) and *IL-10* (p < 0.01) after cyclophosphamide treatment in IPEC-J2 cells (Fig. 3b).

### Capric acid alleviates cyclophosphamide-induced oxidative stress in IPEC-J2 cells

**Figure 4:**
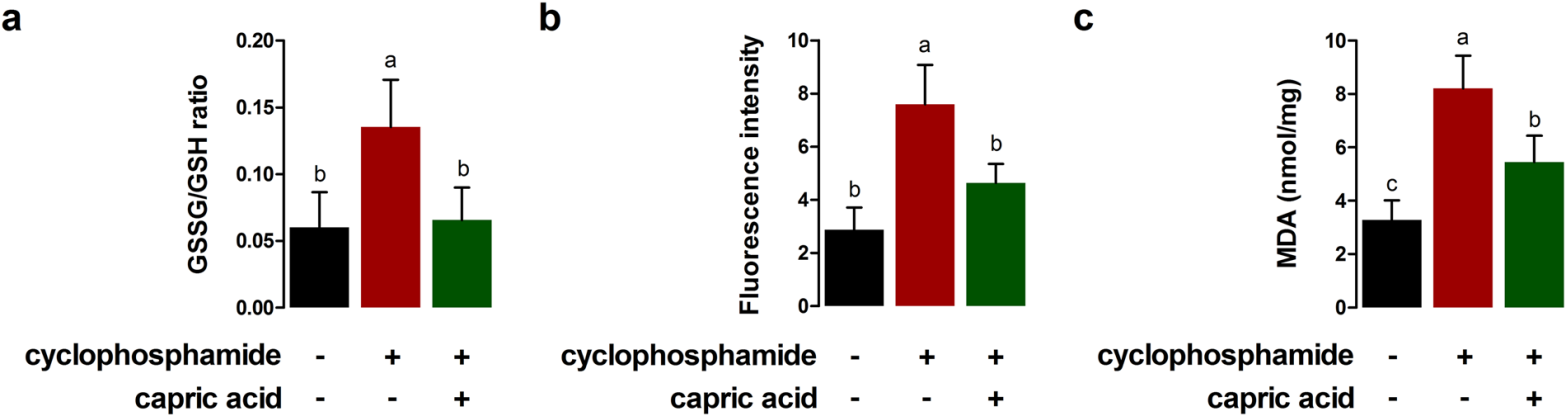
Effects of capric acid treatment on cyclophosphamide-induced oxidative stress in IPEC-J2 cells. (**a**) Glutathione (GSH) and glutathione disulphide (GSSG) concentrations were analysed. (**b**) Intracellular H_2_O_2_ levels were measured. Fluorescence was measured by the Amplex Red method. (**c**) the malondialdehyde (MDA) level was measured by ELISA. Error bars indicate the standard error of the mean (*n* = 3). A p-value of <0.05 was considered to indicate statistical significance. Lowercase letters (**a**,**b**,**c**) indicate significant differences between treatments by the Duncan multiple range tests.

The GSSG/GSH ratio in cyclophosphamide-treated IPEC-J2 cells was significantly higher than that in control cells (p < 0.05) (Fig. 4a). Pre-treatment with capric acid significantly reduced the GSSG/GSH ratio after cyclophosphamide treatment (p < 0.05) (Fig. 4a). To test whether capric acid treatment alleviates ROS production in response to cyclophosphamide-induced oxidative stress in IPEC-J2 cells, the relative extracellular H_2_O_2_ level was monitored (Fig. 4b). Significantly higher H_2_O_2_ levels were observed in IPEC-J2 cells after cyclophosphamide treatment than in control cells (p < 0.05) (Fig. 4b). Pre-treatment with capric acid significantly reduced the extracellular H_2_O_2_ level after cyclophosphamide treatment (p < 0.05) (Fig. 4b). Malondialdehyde levels were significantly higher in cyclophosphamide-treated IPEC-J2 cells than in control cells (p < 0.01) (Fig. 4c). Pre-treatment with capric acid significantly reduced the level of the malondialdehyde after cyclophosphamide treatment (p < 0.05) (Fig. 4c).

**Figure 5:**
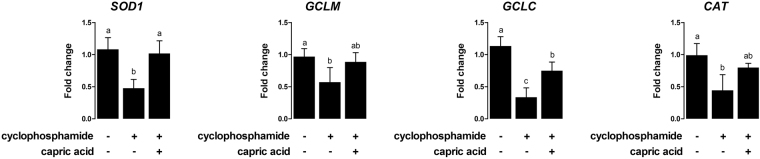
Relative quantitative expression of the genes encoding antioxidant enzymes (SOD1, GCLM, GCLC, and CAT) after capric acid treatment and cyclophosphamide treatment in IPEC-J2 cells. The qRT-PCR data were normalised relative to the expression of *GAPDH* as an endogenous control gene and calculated using the 2^−ΔΔCt^ method (*n* = 3). Error bars indicate the standard error of the mean. A p-value of <0.05 was considered to indicate statistical significance. Lowercase letters (**a**,**b**,**c**) indicate significant differences between treatments based on Duncan multiple range tests.

After cyclophosphamide treatment, relative expression levels of oxidative stress-related genes, such as *SOD1* (p < 0.05), *GCLM* (p < 0.05), *GCLC* (p < 0.01), and *CAT* (p < 0.05), were significantly lower compared to the expression levels in control cells (Fig. 5). Pre-treatment with capric acid significantly increased the expression of *SOD1* (p < 0.05) and *GCLC* (p < 0.05) after cyclophosphamide treatment in IPEC-J2 cells (Fig. 5).

### Capric acid affects intestinal barrier function in cyclophosphamide-treated IPEC-J2 cells

**Figure 6:**
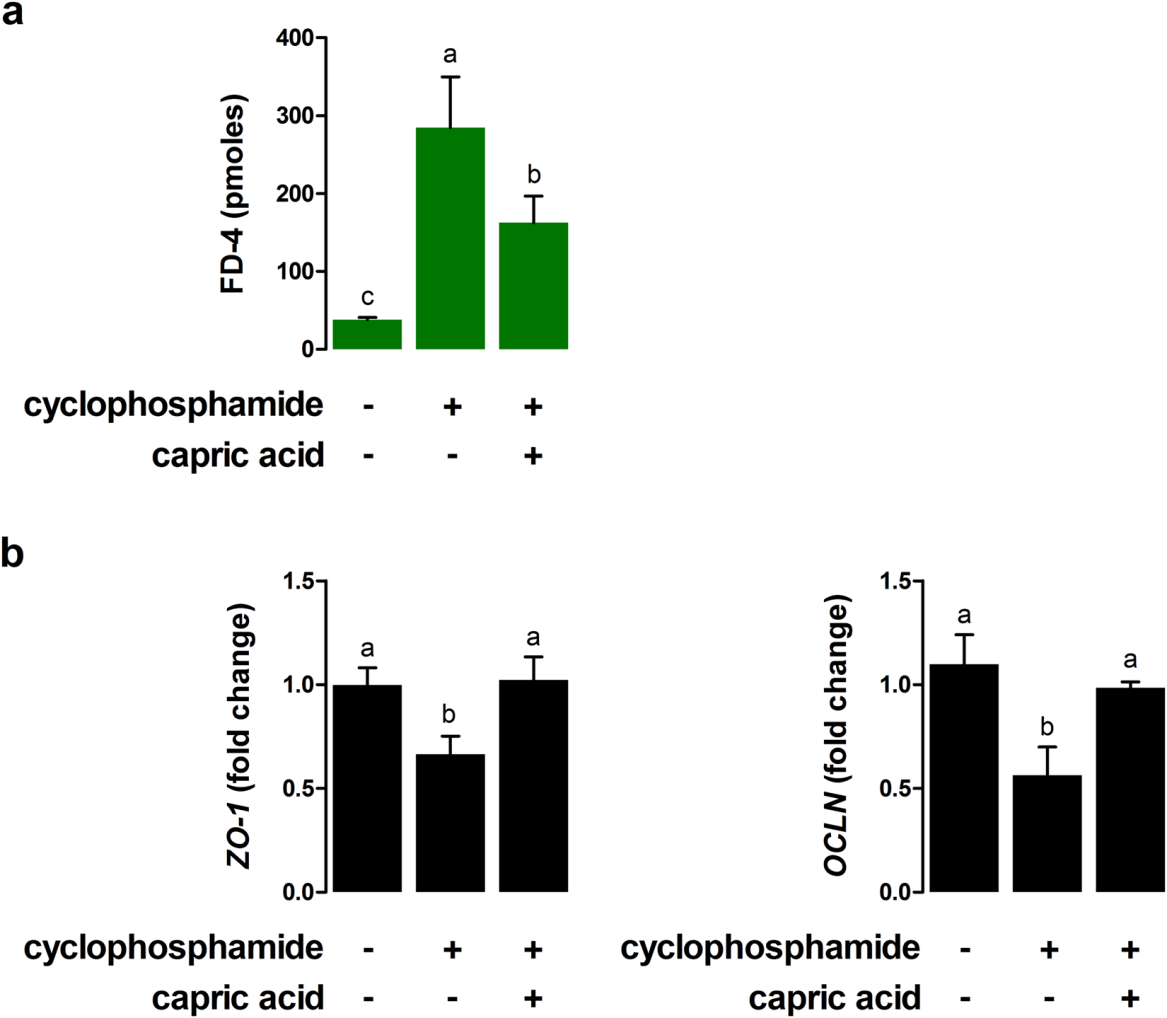
Effects of capric acid treatment on intestinal permeability after cyclophosphamide treatment in IPEC-J2 cells. (**a**) Membrane FD-4 permeability of cyclophosphamide-induced IPEC-J2 cells with or without capric acid pre-treatment. Membrane permeability was quantitated by measuring the amount of FD-4 that permeated the IPEC-J2 monolayer. (**b**) Relative quantitative expression of the genes encoding tight junctions (*ZO-1* and OCLN) for capric acid treatment after cyclophosphamide treatment in IPEC-J2 cells. The qRT-PCR data were normalised to the expression of *GAPDH* as an endogenous control gene and calculated using the 2^−ΔΔCt^ method. Error bars indicate the standard error of the mean (*n* = 3). A p-value of <0.05 indicated statistical significance. Lowercase letters (a, b, c) indicate significant differences between treatments based on Duncan multiple range tests.

To test whether capric acid treatment affects intestinal barrier function in cyclophosphamide-treated IPEC-J2 cells, the permeability of FD-4 was measured. The permeability of FD-4 in cyclophosphamide-treated IPEC-J2 cells was significantly higher than that of the control cells (p < 0.001) (Fig. 6a). Pre-treatment with capric acid significantly reduced the permeability of FD-4 in cyclophosphamide-treated IPEC-J2 cells (p < 0.05) (Fig. 5).

Next, we tested whether capric acid affects tight junction complexes on cyclophosphamide-treated IPEC-J2 cells (Fig. 6b). The mRNA expression levels of *ZO-1* and *OCLN* were significantly lower in cyclophosphamide-treated IPEC-J2 cells than in control cells (p < 0.05) (Fig. 6b). Pre-treatment with capric acid significantly increased the expression of *ZO-1* and *OCLN* in cyclophosphamide-treated IPEC-J2 cells (p < 0.05) (Fig. 6b).

### *In vivo* effect of capric acid on inflammatory and oxidative stress markers in blood serum in cyclophosphamide-treated pigs

**Figure 7:**
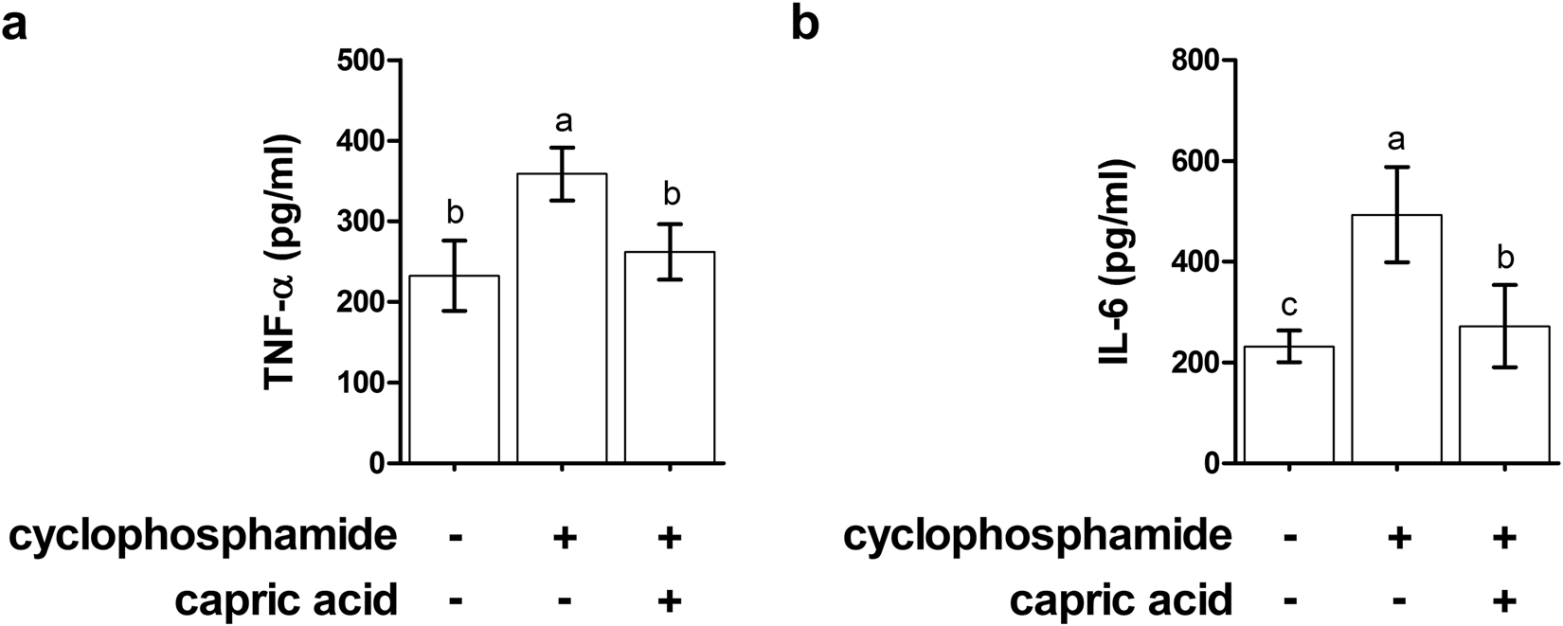
Effects of capric acid on the production of the inflammatory cytokines TNF-α and IL-6 in the blood serum after the CTX challenge. Miniature pigs were randomly allocated into three groups: (T1) control diet + saline challenge; (T2) control diet + CTX challenge; and (T3) control diet with 0.5% capric acid + CTX challenge. Concentrations of TNF-α and IL-6 were determined by ELISA (*n* = 5). Error bars indicate the standard error of the mean (*n* = 3). A p-value of <0.05 was considered to indicate statistical significance. Lowercase letters (a, b, c) indicate significant differences between treatments based on Duncan multiple range tests.

To confirm the effect of capric acid on inflammation and oxidative stress observed in cyclophosphamide-treated IPEC-J2 cells *in vitro*, the levels of inflammatory and oxidative stress markers were evaluated in blood serum in cyclophosphamide-treated pigs *in vivo*. The TNF-α concentration was significantly higher in the blood serum of cyclophosphamide-treated pigs than in control pigs (p < 0.05) (Fig. 7a). Dietary treatment with capric acid significantly reduced the TNF-α concentration in the blood serum in cyclophosphamide-treated pigs (p < 0.05) (Fig. 7a). Additionally, a significantly higher IL-6 concentration was observed in the blood serum of cyclophosphamide-treated pigs than in control pigs (p < 0.05) (Fig. 7b). Dietary treatment with capric acid significantly reduced the IL-6 concentration in the blood serum of cyclophosphamide-treated pigs (p < 0.05) (Fig. 7b).

**Figure 8:**
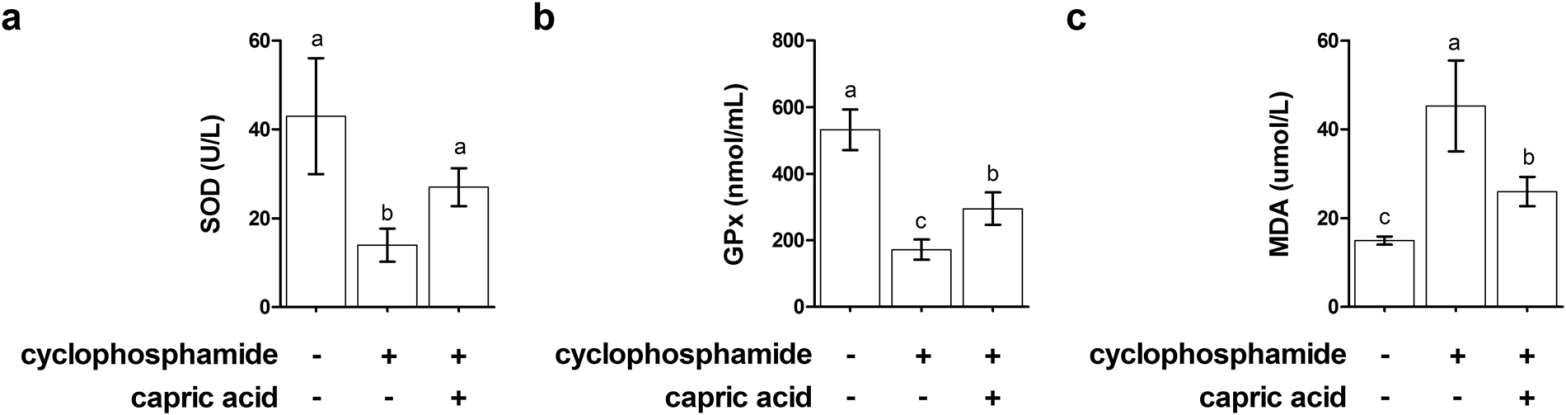
Effects of capric acid on SOD (**a**), GPx (**b**), and MDA (**c**) levels in the blood serum after the CTX challenge. Miniature pigs were randomly allocated into three groups: (T1) control diet + saline challenge; (T2) control diet + CTX challenge; and (T3) control diet with 0.5% capric acid + CTX challenge. Concentrations of SOD, GPx, and MDA were determined by ELISA (n = 5). Error bars indicate the standard error of the mean (*n* = 3). A p-value of < 0.05 was considered to indicate statistical significance. Lowercase letters (a, b, c) indicate significant differences between treatments based on Duncan multiple range tests.

The SOD level in the blood serum of cyclophosphamide-treated pigs was significantly lower than that of control pigs (p < 0.05) (Fig. 8a). Dietary treatment with capric acid significantly increased the SOD level in the blood serum of cyclophosphamide-treated pigs (p < 0.05) (Fig. 8a). GPx activity was significantly lower in the blood serum of cyclophosphamide-treated pigs than in control pigs (p < 0.05) (Fig. 8b). Dietary treatment with capric acid significantly increased the GPx activity in the blood serum of cyclophosphamide-treated pigs (p < 0.05) (Fig. 8b). The malondialdehyde level was significantly higher in the blood serum of cyclophosphamide-treated pigs than in control pigs (p < 0.01) (Fig. 8c). Dietary treatment with capric acid significantly reduced the malondialdehyde level in the blood serum of cyclophosphamide-treated pigs (p < 0.05) (Fig. 8c).

### *In vivo* effects of capric acid on mRNA expression in cyclophosphamide-treated pigs

**Figure 9:**
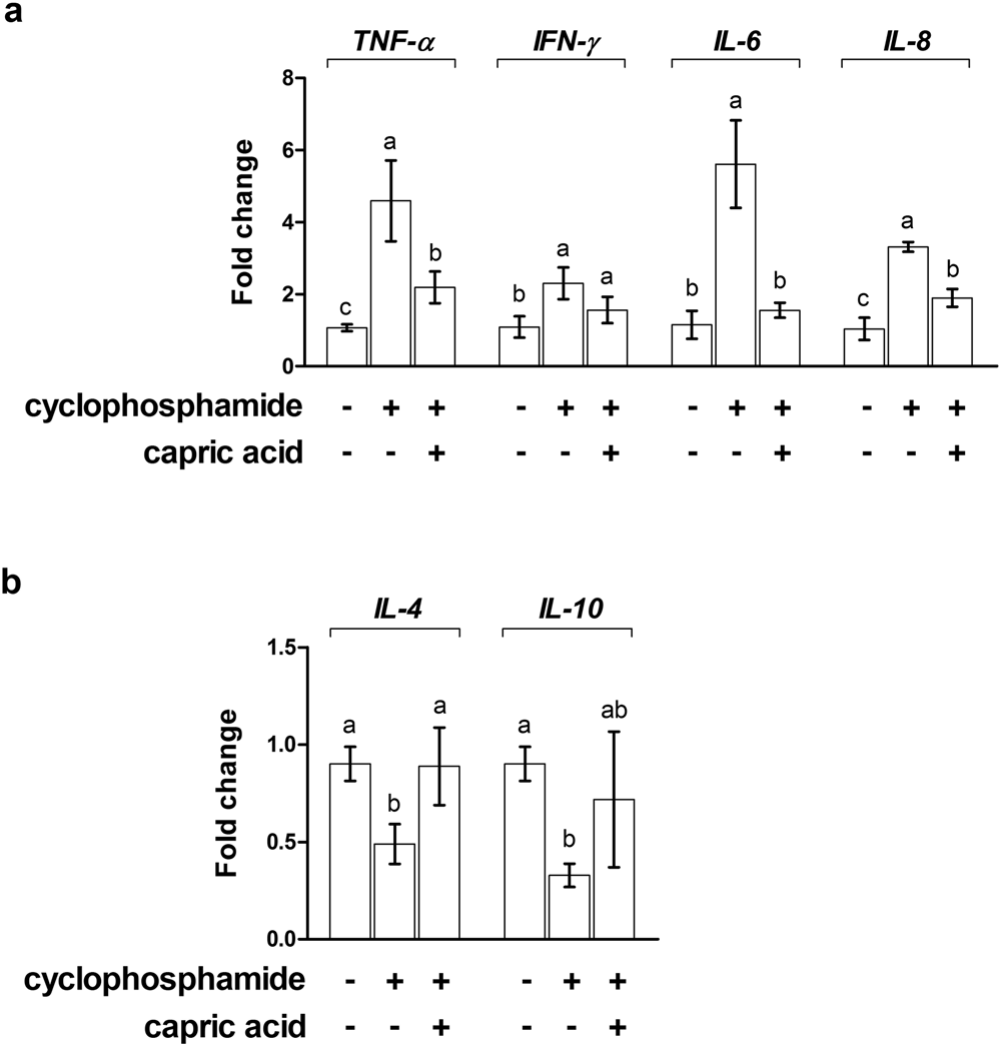
Relative quantitative expression of genes encoding (**a**) pro-inflammatory cytokines (TNF-α, IFN-γ, and IL-6) and (**b**) anti-inflammatory cytokines (IL-4 and IL-10) for capric acid treatment after cyclophosphamide challenge in peripheral blood mononuclear cells of miniature pigs. Miniature pigs were randomly allocated into three groups: (T1) control diet + saline challenge; (T2) control diet + CTX challenge; and (T3) control diet with 0.5% capric acid + CTX challenge. The qRT-PCR data were normalised relative to the expression of *GAPDH* as an endogenous control gene and calculated using the 2^−ΔΔCt^ method. Error bars indicate the standard error of the mean. A p-value of < 0.05 was considered to indicate statistical significance. Lowercase letters (a, b, c) indicate significant differences between treatments based on Duncan multiple range tests.

We examined the expression of mRNAs related to inflammation and oxidative stress in peripheral blood mononuclear cells and those related to intestinal barrier function in the jejunum of the small intestine in cyclophosphamide-treated pigs. The relative expression levels of pro-inflammatory genes, such as *TNF-α* (p < 0.01), *IFN-γ* (p < 0.01), *IL-6* (p < 0.01), and IL-8 (p < 0.01) were significantly higher and those of anti-inflammatory genes, such as *IL-6* (p < 0.05) and IL-8 (p < 0.01) were significantly lower in cyclophosphamide-treated pigs than in control pigs (Fig. 9a and b). Dietary treatment with capric acid significantly reduced the expression of *TNF-α* (p < 0.05), *IL-6* (p < 0.01), and *IL-8* (p < 0.05) and increased the expression of *IL-4* (p < 0.05) in peripheral blood mononuclear cells of cyclophosphamide-treated pigs (Fig. 9a and b).

**Figure 10:**
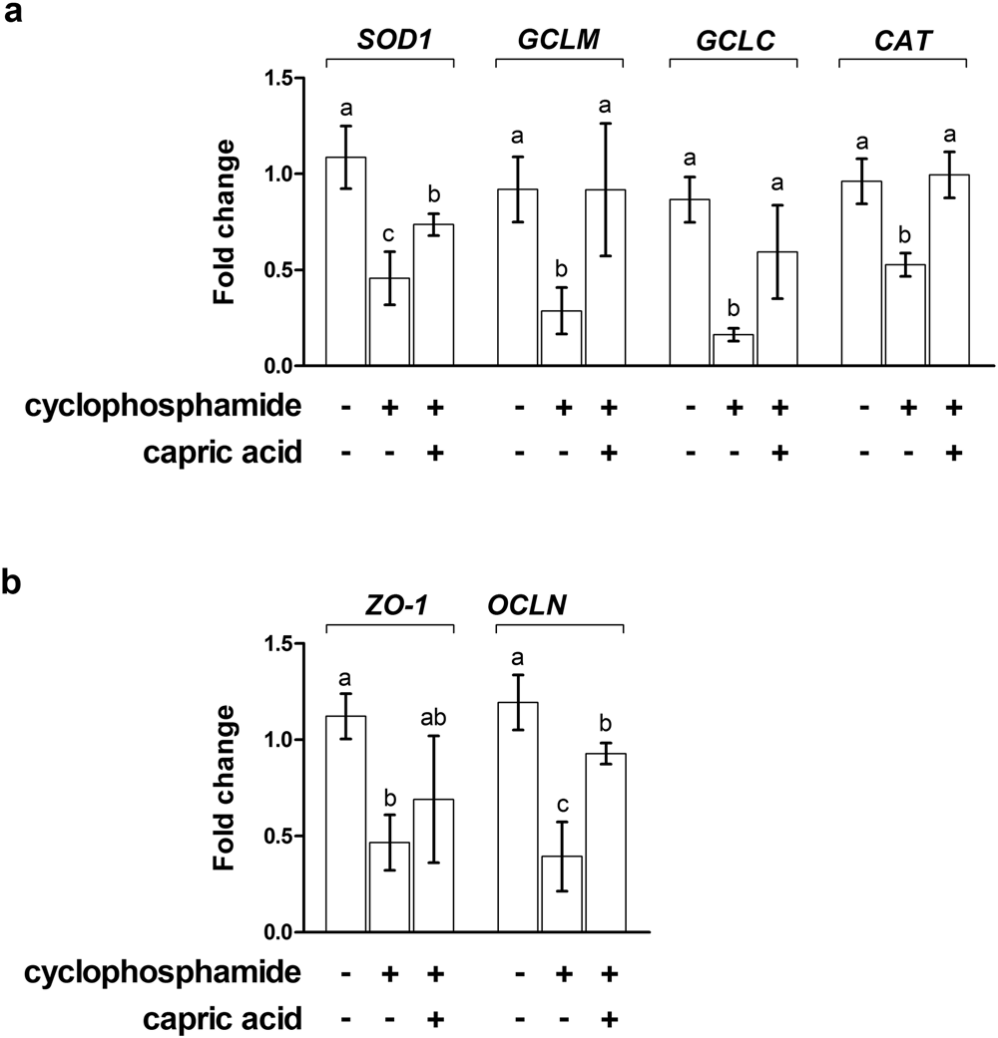
Relative quantitative expression of genes encoding (**a**) antioxidant enzymes (SOD1, GCLM, GCLC, and CAT) and (**b**) tight junctions (ZO-1 and OCLN) after capric acid treatment and cyclophosphamide challenge in peripheral blood mononuclear cells of miniature pigs. The miniature pigs were randomly allocated into three groups: (T1) control diet + saline challenge; (T2) control diet + CTX challenge; and (T3) control diet with 0.5% capric acid + CTX challenge. The qRT-PCR data were normalised relative to the expression of *GAPDH* as an endogenous control gene and calculated using the 2^−ΔΔCt^ method. Error bars indicate the standard error of the mean. A p-value of <0.05 was considered to indicate statistical significance. Lowercase letters (**a**,**b**,**c**) indicate significant differences between treatments based on Duncan multiple range tests.

In cyclophosphamide-treated pigs, the relative expression levels of oxidative stress-related genes, such as *SOD1* (p < 0.05), *GCLM* (p < 0.05), *GCLC* (p < 0.01), and *CAT* (p < 0.01), were significantly lower than those in control pigs (Fig. 10a). Dietary treatment with capric acid significantly increased the expression of *SOD1* (p < 0.05), *GCLM* (p < 0.05), *GCLC* (p < 0.05), and CAT (p < 0.05) in peripheral blood mononuclear cells of cyclophosphamide-treated pigs (Fig. 10a). The relative expression levels of intestinal barrier function-related genes, such as *ZO-1* (p < 0.05) and *OCLN* (p < 0.05), were significantly lower in cyclophosphamide-treated pigs than in control pigs (Fig. 10b). Dietary treatment with capric acid significantly increased the expression of *OCLN* (p < 0.01) in the jejunum in cyclophosphamide-treated pigs (Fig. 10b).

## Discussion

We found that capric acid, an MCFA, significantly reduced the TNF-α and IL-6 concentrations after cyclophosphamide treatment in porcine intestinal epithelial cells. Fatty acids are an important energy source and improve intestinal heath by inhibiting the over-release of intestinal inflammatory mediators, especially pro-inflammatory cytokines, in pigs^8^. Fatty acids could affect inflammatory cell function and inflammatory processes by a variety of general mechanisms. NFκB is a transcription factor involved in the upregulation of inflammatory cytokines including TNFs and ILs^26^. According to a previous report, capric acid inhibits inflammatory cytokines such as IL-8, IL-6, and TNF-α by the inhibition of MAPK phosphorylation and NF-kB activation^24^. The authors elucidated the mechanism by which capric acid attenuates cytokine production and reported that capric acid at 100 mM significantly suppresses phosphorylated MAPKs, such as p38, JNK, and ERK, and significantly increased NF-kB p65 translocation^24^. Additionally, PPAR-γ is a transcription factor with anti-inflammatory functions. Capric acid can regulate inflammatory gene expression and it interferes with the activation of NFκB creating an intriguing interaction between these two transcription factors^27^. A previous report suggested that capric acid is a modulating ligand for PPARs^28^. They demonstrated that capric acid occupies a novel binding site and only partially stabilizes the AF-2 helix of PPARα and binds to PPARα and PPARβ/δ^28^. These observations suggest that capric acid influences inflammatory gene expression via the inhibition of the activation of NFκB and PPARs.

MCFAs occur naturally as medium-chain triglycerides in milk fat and various feed materials; they have specific nutritional effects and can be utilised directly by enterocytes for energy production and thereby support the integrity of the intestine in young piglets^21^. According to a previous report, capric acid enhances IL-8 production in human intestinal epithelial cells (Caco-2), influencing cell function via cellular PKC activity^29^.

Fatty acids induce porcine host defence peptide gene expression in IPEC-J2 intestinal epithelial cells; they improve intestinal morphology, reduce the total viable counts of proximal colon *Clostridium* and *Escherichia coli*, and decrease TNF-α and IL-6 levels in the serum and DNA-binding activity of intestinal nuclear factor-κB in pigs^30^. Taken together, MCFAs, such as capric acid, attenuate intestinal inflammation and promote intestinal health in pigs.

In the present study, capric acid treatment alleviated oxidative stress induced by cyclophosphamide in small intestinal epithelial cells. Management and nutritional strategies have been developed to maximise growth performance and livestock health by considering GI health. The intestinal epithelium plays critical roles in nutrient absorption, the mucosal immune response to pathogenic bacteria, and the regulation of mucosal tissue homeostasis^31^. Because the GI tract comprises more than 70% of the immune cells in the body, the activation of the GI immune system is directly related to livestock health^1^. The small intestine is vulnerable to damage induced by toxins, such as pathogens and toxic chemicals, which affect plasma and intracellular ROS production, resulting in apoptosis and reducing antioxidative capacity and mitochondrial dysfunction^32–34^. The small intestinal epithelial cell is the main target of harmful factors and stress, including toxins and ROS^35^. The imbalance between ROS and antioxidants induces oxidative stress, resulting in the retardation of growth in livestock^36^. According to a previous study, MCFA (caprylic, capric, and lauric)-rich rice bran oils ameliorate arsenite-induced oxidative stress in rats^37^.

The intestinal epithelium has two main functions, i.e. the traffic of nutrients from the lumen and the restriction of the passage of potentially harmful microorganisms and toxins as an intestinal epithelial barrier or paracellular permeability mechanism^38,39^. In the present study, we focused on the epithelial barrier function of capric acid. To maintain the epithelial barrier, effective intercellular junctions are important. Paracellular permeability of harmful microorganisms and toxins is regulated primarily by epithelial intercellular junctions, such as the tight junctions, adherens junctions, and desmosomes^40^. Among epithelial intercellular junctions, adherens junctions and desmosomes are critical for the maintenance of the proximity between epithelial cells via intercellular molecular connections, whereas tight junctions play a role in sealing the paracellular space^41^. In the present study, cyclophosphamide increased the permeability of fluorescent dextran (40 kDa), whereas capric acid treatment restored impaired epithelial barrier function in small intestinal epithelial cells. Additionally, capric acid treatment increased the expression of tight junction-related genes, such as *ZO-1* and *OCLN*, which decreased in response to cyclophosphamide treatment^42^. In agreement with the results of this study, nutrients, such as butyrate, amino acids, and vitamins, play critical roles in intestinal permeability and integrity, as well in the paracellular permeability responsible for allowing the absorption of nutrients and other macromolecules^43–45^. These finding suggested that crosstalk between nutrients and epithelial barrier function occurred by the dynamic regulation of the tight junction and enhance intestinal barrier function via nutritional manipulation.

The pig is a major animal model used in nutritional and translational research and is an alternative to the dog or monkey as a non-rodent animal model in the toxicological testing of pharmaceuticals^46^. In the present study, we investigated the functions of capric acid in cyclophosphamide-treated small intestinal epithelial cells *in vitro* and used miniature pigs as a non-rodent animal model to investigate the function of capric acid in the small intestine. Both humans and minipigs are omnivores and accordingly have similarities with respect to the physiological function of the GI system^46,47^. The small intestinal system of minipigs offers some anatomical and functional advantages, such as absorption and metabolism, compared to other non-rodent animal models for *in vivo* testing. In the present study, 0.5% capric acid was used, which is much higher than the amount used in a previous study (0.2% capric acid) showing a dramatic effect of capric acid *in vivo* ^25^. The previous study reported that 0.2% capric acid supplementation improved piglet performance and the structure of the ileum^25^. Therefore, to determine the appropriate dose of capric acid *in vivo*, further analyses are needed.

In conclusion, it has been reported that MCFAs support the integrity of the intestine, increasing the length of villi and reducing the crypt depth in the small intestine. However, owing to differences in the effects of each MCFA, it is important to study the effect on each fatty acid, such as capric acid, on the intestinal epithelium and the integrity of the intestine. Therefore, in the present study, the effects of a MCFA, capric acid, on intestinal oxidative stress, inflammation, and barrier function were examined in porcine epithelial cells and miniature pigs after treatment with the immune suppressant cyclophosphamide. Capric acid alleviated inflammatory cytokine production (TNF-α and IL-6) and related gene expression (*NF-κB*, *TNF-α*, *IFN-γ*), alleviated oxidative stress (GSSG/GSH ratio, H_2_O_2_, and malondialdehyde), and increased oxidative stress-related gene expression (*SOD1* and *GCLC*) in cyclophosphamide-treated IPEC-J2 cells. Furthermore, the permeability of FD-4 and expression of *ZO-1* and *OCLN* in cyclophosphamide-treated IPEC-J2 cells were reduced by capric acid. Furthermore, dietary capric acid reduced TNF-α, IL-6, and MDA levels and increased SOD, GPx, and the expression of genes related to pro-inflammatory, oxidative stress, and intestinal barrier functions in cyclophosphamide-treated miniature pigs. Our results demonstrated that capric acid improves protection against cyclophosphamide-induced intestinal inflammation, oxidative stress, and barrier function in porcine small intestinal epithelial cells *in vitro* and miniature pigs *in vivo*. Our data improve our general understanding of the functions of capric acid in the small intestine of pigs.

## Methods

### Cell line and culture conditions

The non-transformed porcine intestinal epithelial cell line (IPEC-J2; DSMZ, Braunschweig, Germany), originally isolated from jejunal epithelia of a neonatal unsuckled piglet, were cultured in Dulbecco’s modified Eagle medium (DMEM) and Ham’s F-12 medium mixed at a 1:1 ratio (Gibco Life Technologies, Grand Island, NY, USA) supplemented with 5% foetal bovine serum, 1% insulin-transferrin-selenium-X, and 1% (v/v) penicillin-streptomycin mixture^48^. Cells were grown at 37 °C in a humidified atmosphere of 5% CO_2_.

IPEC-J2 cells were incubated with various concentrations of capric acid (Sigma–Aldrich, Seoul, Korea) for 24 h before cyclophosphamide induction. For cyclophosphamide induction, the IPEC-J2 cells were incubated with various concentrations of cyclophosphamide for 1 h. Cyclophosphamide was removed by washing twice with PBS.

### Analyses of TNF-α, IL-6, GSSG/GSH ratio, intracellular H_2_O_2_, and malondialdehyde levels *in vitro*

After 1 h of treatment with cyclophosphamide, IPEC-J2 cells were incubated with fresh cell culture medium. Culture media were collected after 12 h. The samples were centrifuged (245 × *g*, 10 min) and cytokine concentrations were measured. The levels of TNF-α, IL-6, and malondialdehyde secretion were determined using Porcine-specific Enzyme-linked Immunosorbent Assay (ELISA) Kits (Thermo Scientific, Waltham, MA, USA) according to the manufacturer’s instructions.

To measure the GSSG/GSH ratio, IPEC-J2 cells were pre-treated with or without capric acid for 24 h followed by cyclophosphamide treatment for 1 h using the GSH/GSSG-Glo Glutathione Assay (Promega, Madison, WI, USA). Cells cultured in a 96-well culture plate were harvested after 24 h of treatment by the removal of the cell culture medium, followed by immediate lysis and assays for total and oxidised GSH, following the manufacturer’s instruction.

To measure ROS, the level of intracellular H_2_O_2_ was analysed using the Amplex Red Hydrogen Peroxide Assay Kit (Invitrogen, Molecular Probes, Eugene, OR, USA). IPEC-J2 cells were pre-treated with or without capric acid for 24 h, followed by cyclophosphamide treatment for 1 h in phenol red-free DMEM. The H_2_O_2_ concentrations in the medium were determined using the working solution of 100 μM Amplex Red reagent and 0.2 U/mL horseradish peroxidase. H_2_O_2_ determination was also performed. After 60 min of incubation with the dye at 25 °C H_2_O_2_ was quantitatively analysed; the excitation wavelength was set at 560 nm and emission was measured at 590 nm (Victor × 2 2030 Fluorometer; Perkin Elmer, Waltham, MA, USA).

### Permeability assay

When the IPEC-J2 monolayer was confluent (≥1 kΩcm²), the cells were treated with or without capric acid for 24 h. The cells were washed twice and incubated with cyclophosphamide for 1 h. The cells were washed twice again. The permeability assay started when 500 μL of culture medium containing 50 μg of FD-4 (Sigma-Aldrich) was added to the apical chamber. The basolateral chamber was filled with 1.5 mL of culture medium (37 °C, 5% CO_2_). FD-4 was allowed to permeate overnight (18 h) from the apical to the basolateral chamber. Subsequently, 100 μL of the basolateral chamber medium was transferred to a 96-well plate to measure the amount of permeated FD-4 using a flour-spectrophotometer (Ex/Em: 490/520 nm).

### *In vivo* cyclophosphamide challenge in pigs

All experiments were approved by the Animal Care Committee of Dankook University and were conducted in accordance with the guidelines for the care and use of experimental animals for research at Dankook University. A total of 15 miniature pigs [MK strain, (Duroc × Yorkshire) × (Pot Valley × Berkshire) × Yucatan] with an average initial body weight of 20.92 ± 0.24 kg were used to evaluate the effects of dietary capric acid over a 21-day period. Each pig was kept in an individual pen and housed in an environmentally controlled nursery facility with slatted plastic flooring and a mechanical ventilation system. Each room was maintained at approximately 27 °C and 60% humidity. Each pen was equipped with a one-sided, stainless steel self-feeder and a nipple drinker, which allowed *ad libitum* access to feed and water. Experimental treatments were as follows: (T1) control diet + saline challenge, (T2) control diet + CTX challenge, and (T3) control diet with 0.5% capric acid + CTX challenge. The control diet was based on corn and soybean meal.

For the CTX challenge assay, all pigs from each dietary treatment group were injected intraperitoneally with CTX or a saline solution at day 14. CTX (Sigma–Aldrich) was diluted in a sterile saline solution and injected at 0.01% (50 mg/kg) of the body weight on the 14th day after the feeding trial. The dose of CTX was determined based on the results of a previous study^49^. No vaccines or antibiotics were used in this experiment.

### Blood collection and biochemical analysis

At the end of the experiment (21st day), blood samples were collected and analysed according to our standard protocol^50^. Briefly, blood samples were collected from all pigs via jugular venipuncture 6 h after the challenge into a non-heparinised K_3_EDTA vacuum tube (Becton Dickinson Vacutainer Systems, Franklin Lakes, NJ, USA) to obtain serum and whole blood. Leukocyte, lymphocyte, and monocyte counts were determined using an automatic blood analyser (ADVIA 120; Bayer, Leverkusen, Germany).

The whole blood samples were subsequently centrifuged at 3,000 × *g* for 15 min at 4 °C, and the serum was harvested. Thereafter, the samples were frozen and stored at −20 °C until further analysis. The levels of serum TNF-α (R&D Systems, Minneapolis, MN, USA), IL-6 (R&D Systems), superoxide dismutase (SOD) (Cohesion Biosciences, London, UK), glutathione peroxidase (GPx) (Cayman Chemical, Ann Arbor, MI, USA), and malondialdehyde (MDA) (Abcam, Cambridge, UK) were determined using an ELISA kit.

### Peripheral blood mononuclear cell preparation

For PBMC isolation, blood samples (5–10 mL) from 15 pigs were collected in a K_3_EDTA vacuum tube at the end of the experiment. PBMCs were prepared according to a previous study^51^. Briefly, the collected blood samples were diluted with an equal volume of a balanced salt solution, and PBMCs were immediately isolated by Histopaque density gradient centrifugation according to the manufacturer’s instructions (Sigma–Aldrich). Briefly, the diluted blood samples were mixed with a half volume of a Histopaque solution and then centrifuged at 400 × *g* for 35 min at room temperature. PBMCs were carefully aspirated from the Histopaque solution–plasma interface.

### Quantitative real-time polymerase chain reaction

RNA was isolated using TRIzol reagent (Invitrogen, Carlsbad, CA, USA). For quantitative real-time polymerase chain reaction (RT-qPCR), total RNA (0.1–1 µg) was used for complementary DNA synthesis using the Maxima First-strand cDNA Synthesis Kit (Life Technologies). The primers for RT-qPCR for each gene transcript were designed using Primer3 (http://frodo.wi.mit.edu/) (Table 1). RT-qPCR was performed using a 7500 Fast Real-time PCR System (Applied Biosystems). The RT-qPCR conditions were as follows: 94 °C for 3 min, followed by 40 cycles at 94 °C for 30 s, 59–61 °C for 30 s, and 72 °C for 30 s. Melting curve profiles were analysed for the amplicons. RT-qPCR data were normalised relative to the expression of glyceraldehyde 3-phosphate dehydrogenase (*GAPDH*), an endogenous control gene, and calculated using the 2^−ΔΔCt^ method, where ΔΔCt (cycle threshold) = ΔCt (treated) − ΔCt (control) and ΔCt = Ct of the target gene − Ct of *GAPDH* (treated or control, respectively)^52^.

**Table 1:** List of primers.

**Gene symbol**	**Description**	**Accession No**.	**Forward (5′-> 3′)**	**Reverse (5′->3′)**
*TNF-α*	Tumor necrosis factor alpha	NM_214022	TCTCCTTCCTCCTGGTCGCA	TCCCTCGGCTTTGACATTGG
*IFN-γ*	Interferon gamma	NM_213948	GGCCATTCAAAGGAGCATGG	GATGGCTTTGCGCTGGATCT
*NF-κB*	Nuclear factor of kappa light polypeptide gene enhancer in B-cells 1	NM_001048232	GACAACATCTCCTTGGCGGG	TCTGCTCCTGCTGCTTTGAGG
*IL-4*	Interleukin-4	NM_214123	TCCACGGACACAAGTGCGAC	TGTTTGCCATGCTGCTCAGG
*IL-6*	Interleukin-6	NM_214399	AGCCCACCAGGAACGAAAGA	AGCCATCACCAGAAGCAGCC
*IL-10*	Interleukin-10	NM_214041	CATCCACTTCCCAACCAGCC	CTCCCCATCACTCTCTGCCTTC
*SOD1*	Superoxide dismutase 1	NM_001190422	GTACCAGTGCAGGTCCTCAC	TTTGCCAGCAGTCACATTGC
*GCLM*	Glutamate-cysteine ligase modifier subunit	XM_001926378	TTGGAGCAGCTGTACCAGTG	GAGCTTCCTGGAAACTCGCT
*GCLC*	Glutamate-cysteine ligase catalytic subunit	XM_003482164	GTCCAGTTGGTCCTGTCTGG	CGGGAGTCCCTTCGATCATG
*CAT*	Catalase	NM_214301	ACACAGGCACATGAACGGAT	GTCCCGGATGCCATAGTCAG
*ZO-1*	Zonula occludens 1	XM_021098856	GATCCTGACCCGGTGTCTGA	TTGGTGGGTTTGGTGGGTTG
*OCLN*	Occludin	NM_001163647	GAGAGAGTGGACAGCCCCAT	TGCTGCTGTAATGAGGCTGC
*GAPDH*	Glyceraldehyde-3-phosphate dehydrogenase	NM_001206359	AATGGGGTGATGCTGGTGCT	GGCAGAAGGGGCAGAGATGA

### Statistical analysis

Data were analysed with the general linear model (PROC-GLM) procedure of SAS to determine the significance of differences between the treatments. Results are presented as means and the standard error of the mean (n ≥ 3, where n refers to the number of replicate experiments). The individual miniature pigs were considered the experimental unit. A p-value of < 0.05 indicated statistical significance. Significant differences between treatments were assessed by Duncan multiple range tests.
